# Supplementary material for: Salmonella Enteritidis Bacteriophages Isolated from Kenyan Poultry Farms Demonstrate Time-Dependent Stability in Environments Mimicking the Chicken Gastrointestinal Tract
Source: Viruses. 2022 Aug 16;14(8):1788. doi: 10.3390/v14081788 (PMC9416366; doi:10.3390/v14081788)
Supplement: Supplementary file 1 [file viruses-14-01788-s001.zip › Supplementary Figure S5.pdf]

Supplementary Figure S5

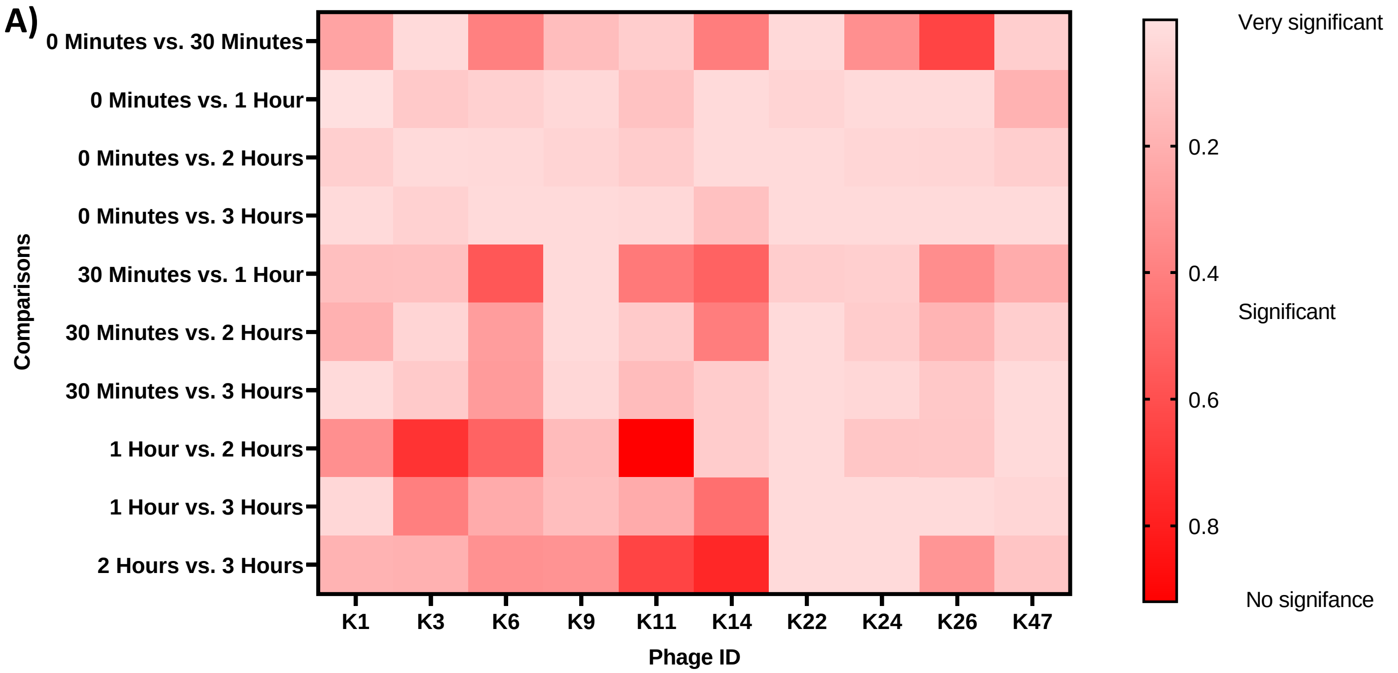

B)

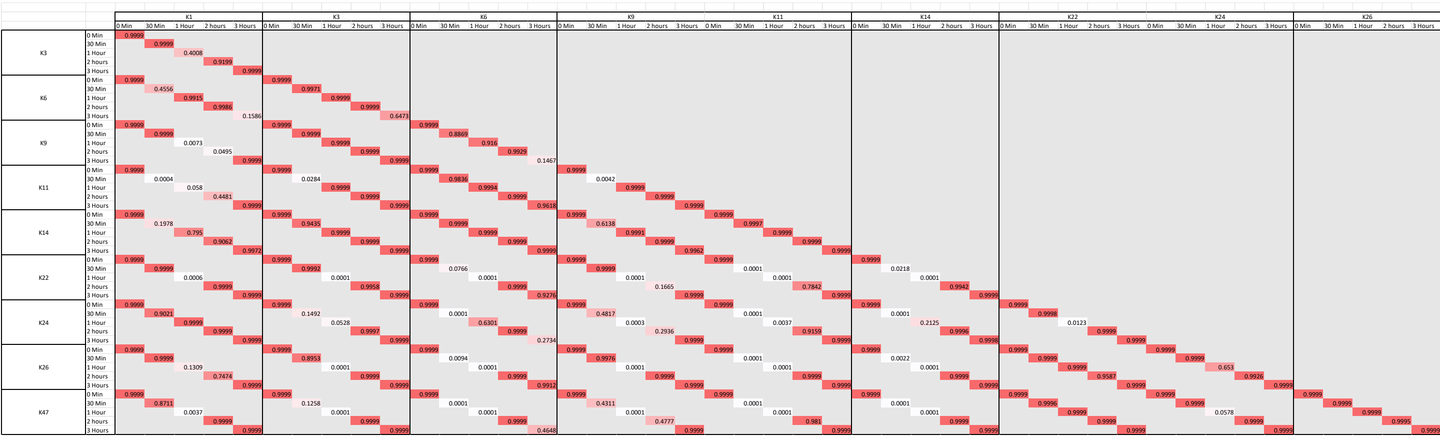

		K1	K3	K6	K9	K11	K14	K22	K24	K26
K3	0 Min	0.9999								
	30 Min	0.9999								
	1 Hour	0.4008								
	2 hours	0.9199								
	3 Hours	0.9999								
K6	0 Min	0.9999	0.9999							
	30 Min	0.4556	0.9971							
	1 Hour	0.9915	0.9999							
	2 hours	0.9986	0.9999							
	3 Hours	0.1586	0.6473							
K9	0 Min	0.9999	0.9999	0.9999						
	30 Min	0.9999	0.9999	0.8869						
	1 Hour	0.0073	0.9999	0.916						
	2 hours	0.0495	0.9999	0.9929						
	3 Hours	0.9999	0.9999	0.1467						
K11	0 Min	0.9999	0.9999	0.9999	0.9999					
	30 Min	0.0004	0.0284	0.9836	0.0042					
	1 Hour	0.058	0.9999	0.9994	0.9999					
	2 hours	0.4481	0.9999	0.9999	0.9999					
	3 Hours	0.9999	0.9999	0.9618	0.9999					
K14	0 Min	0.9999	0.9999	0.9999	0.9999	0.9999				
	30 Min	0.1978	0.9435	0.9999	0.6138	0.9997				
	1 Hour	0.795	0.9999	0.9999	0.9991	0.9999				
	2 hours	0.9062	0.9999	0.9999	0.9999	0.9999				
	3 Hours	0.9972	0.9999	0.9999	0.9962	0.9999				
K22	0 Min	0.9999	0.9999	0.9999	0.9999	0.9999	0.9999			
	30 Min	0.9999	0.9992	0.0766	0.9999	0.0001	0.0218			
	1 Hour	0.0006	0.0001	0.0001	0.0001	0.0001	0.0001			
	2 hours	0.9999	0.9958	0.9999	0.1665	0.7842	0.9942			
	3 Hours	0.9999	0.9999	0.9276	0.9999	0.9999	0.9999			
K24	0 Min	0.9999	0.9999	0.9999	0.9999	0.9999	0.9999	0.9999		
	30 Min	0.9021	0.1492	0.0001	0.4817	0.0001	0.0001	0.9998		
	1 Hour	0.9999	0.0528	0.6301	0.0003	0.0037	0.2125	0.0123		
	2 hours	0.9999	0.9997	0.9999	0.2936	0.9159	0.9996	0.9999		
	3 Hours	0.9999	0.9999	0.2734	0.9999	0.9999	0.9998	0.9999		
K26	0 Min	0.9999	0.9999	0.9999	0.9999	0.9999	0.9999	0.9999	0.9999	
	30 Min	0.9999	0.8953	0.0094	0.9976	0.0001	0.0022	0.9999	0.9999	
	1 Hour	0.1309	0.0001	0.0001	0.0001	0.0001	0.0001	0.9999	0.653	
	2 hours	0.7474	0.9999	0.9999	0.9999	0.9999	0.9999	0.9587	0.9926	
	3 Hours	0.9999	0.9999	0.9912	0.9999	0.9999	0.9999	0.9999	0.9999	
K47	0 Min	0.9999	0.9999	0.9999	0.9999	0.9999	0.9999	0.9999	0.9999	0.9999
	30 Min	0.8711	0.1258	0.0001	0.4311	0.0001	0.0001	0.9996	0.9999	0.9999
	1 Hour	0.0037	0.0001	0.0001	0.0001	0.0001	0.0001	0.9999	0.0578	0.9999
	2 hours	0.9999	0.9999	0.9999	0.4777	0.981	0.9999	0.9999	0.9999	0.9995
	3 Hours	0.9999	0.9999	0.4648	0.9999	0.9999	0.9999	0.9999	0.9999	0.9999

Supplementary Figure S5. P-values of phage stability in TSB at 37 °C . A) P values of phage titres between time-points for a given phage. The colour intensity correlates with the P-value; deep red represents a high P-value, while light pink represents a low P-value. **B)** P-values of phage titres between different phages at a given time-point. The colour intensity correlates with the P-value; deep red represents a high P-value, while white represents a low P-value.
