# Supplementary material for: Salmonella Enteritidis Bacteriophages Isolated from Kenyan Poultry Farms Demonstrate Time-Dependent Stability in Environments Mimicking the Chicken Gastrointestinal Tract
Source: Viruses. 2022 Aug 16;14(8):1788. doi: 10.3390/v14081788 (PMC9416366; doi:10.3390/v14081788)
Supplement: Supplementary file 1 [file viruses-14-01788-s001.zip › Supplementary Figure S6.pdf]

Supplementary Figure S6

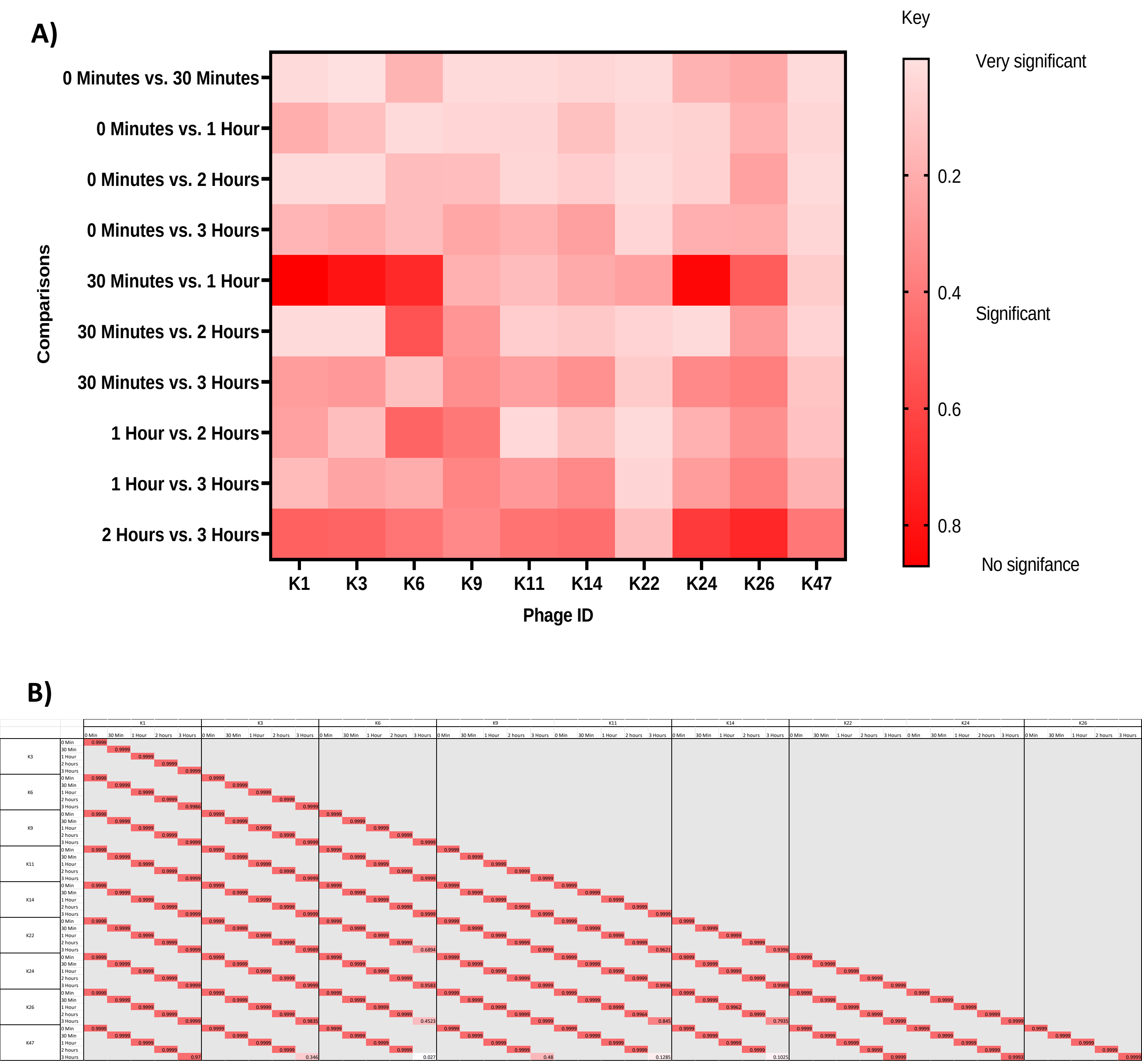

Supplementary Figure S6. P-values of phage stability in TSB at 42 °C . A) P values of phage titres between time-points for a given phage. The colour intensity correlates with the P-value; deep red represents a high P-value, while light pink represents a low P-value. **B)** P-values of phage titres between different phages at a given time-point. The colour intensity correlates with the P-value; deep red represents a high P-value, while white represents a low P-value.
