# Supplementary figures and images for: Salmonella Enteritidis Bacteriophages Isolated from Kenyan Poultry Farms Demonstrate Time-Dependent Stability in Environments Mimicking the Chicken Gastrointestinal Tract
Source: Viruses. 2022 Aug 16;14(8):1788. doi: 10.3390/v14081788 (PMC9416366; doi:10.3390/v14081788)

# Supplementary Figure S2

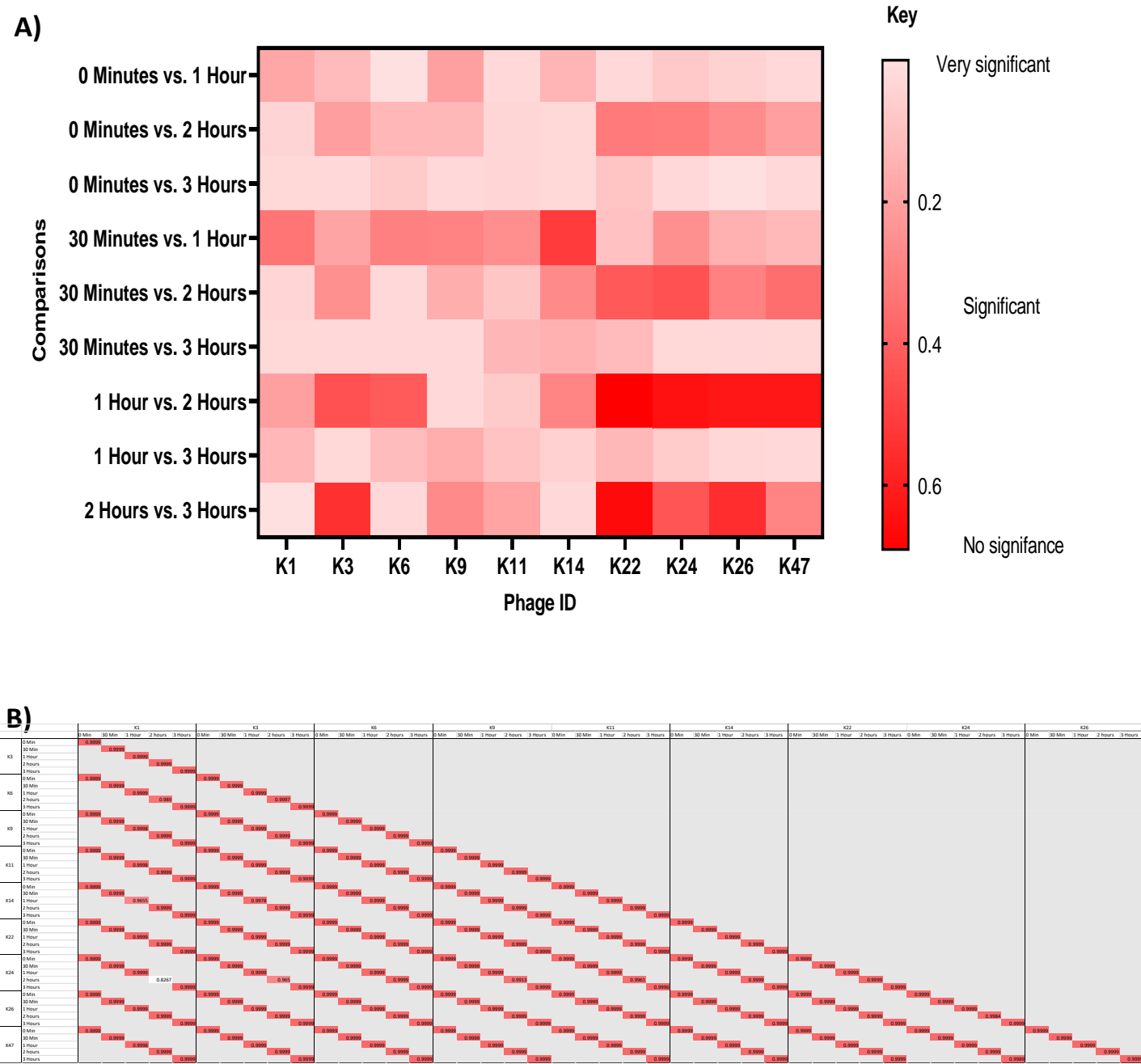

Supplement: Supplementary file 1 [file viruses-14-01788-s001.zip › Supplementary Figure S2.pdf]

Supplementary Figure S8

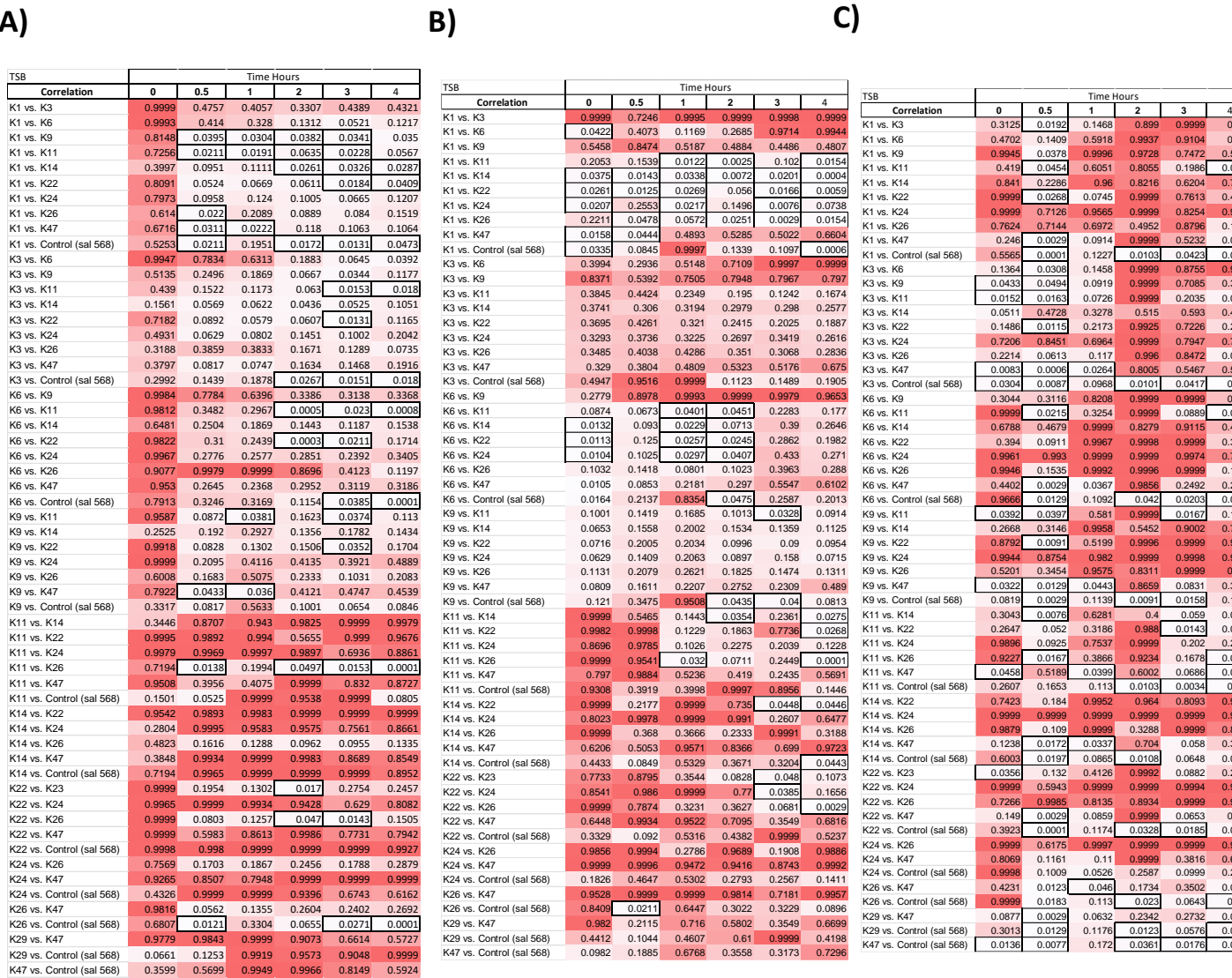

Supplement: Supplementary file 1 [file viruses-14-01788-s001.zip › Supplementary Figure S8.pdf]

# Supplementary Figure S9

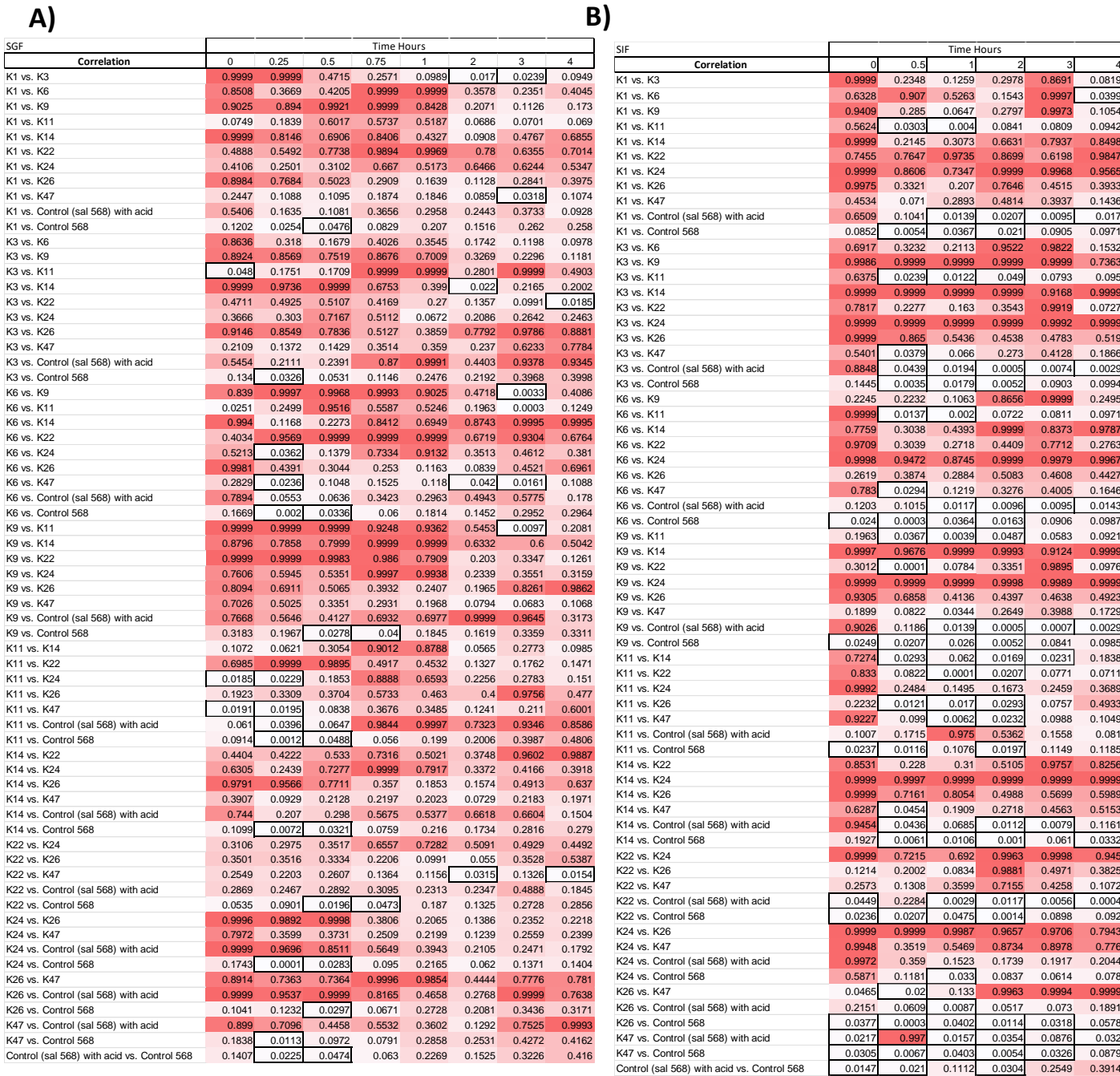

Supplement: Supplementary file 1 [file viruses-14-01788-s001.zip › Supplementary Figure S9.pdf]
